# Mortality and morbidity study of petrochemical employees in a polluted site

**DOI:** 10.1186/1476-069X-11-34

**Published:** 2012-05-18

**Authors:** Roberto Pasetto, Amerigo Zona, Roberta Pirastu, Achille Cernigliaro, Gabriella Dardanoni, Sebastiano Pollina Addario, Salvatore Scondotto, Pietro Comba

**Affiliations:** 1 Department of Environment and Primary Prevention, Istituto Superiore di Sanità, Viale Regina Elena 299, Rome 00161, Italy; 2 Department of Biology and Biotechnologies “Charles Darwin”, Sapienza Università di Roma Piazzale Aldo Moro 5, 00185, Rome, Italy; 3 Epidemiological Observatory, Regional Health Authority, Via Mario Vaccaro 5, Palermo 90145, Italy

**Keywords:** Cohort study, Mortality, Morbidity, Petrochemical industry, Polluted site

## Abstract

**Background:**

The area of Gela was included among the 57 Italian polluted sites of national interest for environmental remediation because of its widespread contamination from a petrochemical complex. The present study investigates mortality and morbidity of the cohort of Gela petrochemical workers with the aim of disentangling occupational from residential risk.

**Methods:**

Mortality was assessed for 5,627 men hired from 1960, year of the plant start-up, to 1993; it was followed up for vital status in the period 1960–2002. Morbidity was analysed for 5,431 workers neither dead nor lost to follow-up from 1960 to 2001 and was based on Hospital Discharge Records in the period 2001–2006. The work experience was classified in terms of job categories such as *blue collars*, *white collars*, and *both* – workers who shifted from blue to white collar (95%) or vice versa. An *ad hoc* mobility model was applied to define qualitative categories of residence in Gela, as *residents* and *commuters*. Standardized Mortality Ratios (SMRs) and Mortality Rate Ratios (MRRs) were computed, the latter by using a Poisson regression model. Morbidity was analyzed in terms of Hospital Discharge Odds Ratios (HDORs) through a logistic regression model. While performing the internal comparisons, *white collars* was the reference category for the job analysis, and *commuters* was the reference category for the residential analysis.

**Results:**

In the light of epidemiological evidence about health risk from petrochemical industries in both occupational and environmental settings, and/or on the basis of information about occupational and residential contamination and health risk in the area of Gela, noteworthy results are shown for lung cancer [MRR: 2.11 (**CI** 90%; 0.96-4.63) in *blue collars*; 1.71 (1.09-2.69) in *residents*], respiratory diseases [HDOR: 2.0 (1.0-3.0) in *blue collars*; 1.4 (0.96-2.06) in *residents*] and genitourinary diseases [HDOR: 1.34 (1.06-1.68) in *blue collars*; 1.23 (1.04-1.45) in *residents*].

**Conclusions:**

The results support a role of the exposures in the occupational and residential settings, the latter due to the local ascertained contamination, in affecting the workers’ health. These results underline the urgent need of water, soil, air and food-chain monitoring programs, to discover active sources of exposure and consequently define public health interventions.

## Introduction

Gela is a town of approximately 80,000 inhabitants located by the sea in the South West coast of Sicily Region, in Southern Italy. Crude oil was found in the underground in the late ‘50s; since the early ‘60s a vast area of Gela municipality, nearby Gela town, has hosted a large oil refinery, together with a thermoelectric power plant and petrochemical plants for production of organic (ethylene, acrylonitrile) and inorganic (sulphuric acid, ammonia, chlorine, urea) chemicals. From 1998, a part of Gela municipal area — the entire petrochemical complex and an extended sea portion — was included among the 57 Italian Polluted Sites (IPSs) of national interest for environmental remediation. Data collected in this site after year 2000 documented groundwater, soil and air contamination. Maximum concentrations of arsenic, vinyl chloride, mercury and benzene were some orders of magnitude higher than the threshold values [1,2]. It was documented that soil and shallow water in Gela IPS were severely contaminated by metals and by organohalogenated compounds [1]. On the basis of the Italian National Registry of Emissions and Sources data [3], the emissions of NOx, SOx, and benzene in Gela area represented respectively 11%, 30%, and 31% of the total emissions occurring in Sicily. Furthermore, two monitoring studies of pine needles and road dust samples showed that Gela town was heavily affected by metals and metalloids [4,5]. Finally, a recent biomonitoring study in a group of Gela residents showed high levels of total arsenic in urine [6]. Urinary arsenic levels higher than reference values, although indicative of recent exposures [7], are of interest because inorganic arsenic or its metabolites may cause adverse health effects including lung cancer [8,9].

In 1970, the case of Gela was described as an example of “industrialization without development” [10], because the expected evolution of local cultural and societal structures, figured on the basis of the capitalistic industrialization in Northern Europe and North America, had not taken place. The situation in Gela recalled the one that can be seen in developing countries, where an industrialization process externally promoted, not deriving from the evolution of the local society, could not induce local territorial and population development [11], but produced environmental contamination. In this context, the implementation of environmental monitoring and reclamation activities can be hampered. In 1995 a Decree called for an air quality monitoring program [12]; still, at the end of 2010 such program was not implemented yet [13]. As far as the health status of residents is concerned, since the mid ‘90s ecological mortality and morbidity studies showing probable health risks from environmental and occupational exposures were conducted [14-16].

After year 2000, the public prosecutor in Gela started an inquiry on potential industrial pollution-related health risks concerning both the workers and the general population. Workers employment rosters were seized, and a mortality study of Gela petrochemical workers was carried out [17,18]. The cohort study was successively endorsed and funded by the WHO European Centre for Environment and Health as part of the Project “Technical assistance to the Sicilian Region, Special Office for risk areas to develop plans for environmental remediation”, in three Sicilian areas at high environmental risk, including Gela. The present paper aims at studying mortality and morbidity of Gela petrochemical workers to disentangle occupational from residential health risks.

## Methods

### Cohort definition

Workers employed in the petrochemical plant in Gela from January 1, 1960, namely from the start of plant operation, to January 31, 2002, were 7,147 (6,961 men, 186 women). The cohort considered in the present study comprised 5,627 workers for the mortality analysis and 5,431 for the morbidity analysis.

In the mortality study, all men hired from 1960 to 1993 and born in Sicily were included. The vital status was ascertained for the period 1960–2002 through a request to the municipalities’ registry office; the workers hired after 1993 were excluded to allow a minimum 10 years latency time for delayed exposures effects [19]. The causes of death were coded by an expert nosologist according to International Classification of Diseases (ICD) ruling in the year of death. The vital status was unknown for 4.1% of the total cohort and the cause of death was missing for 27 deaths (4.6%) in the restricted cohort.

In the morbidity study, 5,431 men hired from 1960 to 2000 not deceased or lost to follow-up, and born in Sicily were included. The Hospital Discharge Records (HDRs) available for the Sicily Region in the period 2001–2006 were analysed; the outcome was hospitalization. For each subject the first hospitalization for any given cause of *a priori* interest was selected; the information on main diagnosis was used in the analysis. The Sicilian HDRs Registry covers all the residents’ hospital admissions in or out of their own Region. For each HDR, the fiscal code (a univocal identification code for Italian citizens) was reconstituted. HDRs containing no valid personal data were excluded. Day-hospital admissions, rehabilitation, long stays and duplicate records were also excluded. HDRs were linked to cohort subjects using fiscal code as a key.

Twenty employment rosters of eight different companies were used to collect workers’ personal and employment data. Data from most of the workers were available in different employment rosters; in each roster, besides personal data, the date of start of employment and the job category in terms of *blue collar* or *white collar* were registered. The majority of workers were employed in different companies inside the complex; the duration of employment could not be determined as the only reliable information was the hiring date, and the termination date of employment was not recorded. No data were available about occupational exposures at individual or group level.

Workers had different origins: 85% of them came from Sicily and approximately 15% from other Regions [18]. The latter were hired chiefly in the first 10 years of plant operation [18], mainly to train unskilled local workers [20], and usually lived in Gela for short periods; then they moved to other places in Italy where some petrochemical plants newly constructed needed skilled labour to start operation [10]. Workers born outside Sicily were excluded from the present study to ensure that Sicilian population was an appropriate comparison in the analysis with an external reference, and to minimize exposure heterogeneity and unknown sources of potential confounding.

### Outcomes of *a priori* interest

Some main diseases groups and single specific diseases, for which the epidemiological evidence had shown an association with occupation in petrochemicals or residence in the neighbourhoods, were analysed [21,22]. The following neoplastic (a) and non-neoplastic (b) diseases were selected: a) malignant neoplasms of stomach, colon-rectum, liver, trachea, bronchus and lung, pleura, skin, bladder, kidney, central nervous system (CNS), Hodgkin disease, non-Hodgkin lymphoma, multiple myeloma, leukemias; b) chronic obstructive pulmonary disease (COPD), asthma, nephritis, nephrotic syndrome and nephrosis.

### Job and residence categories

Workers were classified into one of the following three job categories: *only white collars* if they were registered only as white collar in all the employment rosters*, only blue collars* if they were registered only as blue collar in all the employment rosters, *both white* and *blue collars* if they changed from blue collar to white collar or vice versa in different employment rosters; from now on *white collars*, *blue collars*, *both*.

The reconstruction of the residential history was unfeasible. The companies’ rosters provided information on residence at hiring for about 90% of the workers, but this information was not used as it was specific for a given point in time. Moreover, the hiring might have happened before the official change of residence (and usually firms do not need to update internal documents). The companies’ rosters registered also workers’ birthplace abstracted from the identity document or driving license; the birthplace was available for almost all the workers (98.5%). This information had already been used in a previous study to classify workers as residents in Gela or commuters, but the criterion to define commuters was not robust [18]. In the present study the workers were classified as residents in Gela or commuters using the procedure described as follows.

Firstly, birthplace data were used on the basis of two assumptions: a) the workers born in Gela were likely to have been residents in Gela at least during the employment period; b) the workers born in Sicilian municipalities other than Gela might have been or not been residents in Gela during the employment period. The assumption b) was based on anecdotal information from several documents [10,23] successively confirmed by a qualitative sociological study [11,20].

Secondly, a gravity-type mobility model was adopted to identify commuting workers. The mobility model has been described elsewhere [24]; it was implemented to estimate the probability that a "central locality", where a specific plant is established, attracts commuting flows from other places. The general model was applied to the Gela case, thus defining the probability of commuting to Gela from other Sicilian municipalities [24].

Finally, the mobility model results were applied to define two qualitative categories of likelihood of residence in Gela:

a) *Commuters* - workers born in Sicilian municipalities, excluding Gela with a probability of commuting defined by the model as >=0.5 (N. 3,234);

b) *Residents* - workers born in Gela (N. 1,684) and in Sicilian municipalities with a probability of commuting defined by the model as <0.5 (N. 709). The latter were fixed as “moved to Gela at hiring”.

### Statistical analysis

To compute Standardized Mortality Ratios (SMRs) the expected number of deaths was based on the Sicilian death rates specific for gender, 5-year age and calendar time periods. Mortality data are registered by the Italian National Bureau of Statistics, and are electronically available starting from 1970; therefore the mortality rates for the period 1970–75 were applied to the period 1960–1970. The unknown causes of death contributed to the SMRs calculation; that is, a distribution of unknown causes equal to the known ones was assumed and the number of unknown deaths was added to each group on a proportional basis [19].

For the internal analysis, Mortality Rate Ratios (MRRs) were calculated applying a Poisson regression model using as predictive variables job and residence categories, age, calendar period, and time since first employment. Hospital Discharge Odds Ratios (HDORs) were calculated applying a Logistic regression model, using as predictive variables job and residence categories, age, and time since first employment. *White collars* was the reference category for job and *Commuters* was the one for residence. A multiple imputation strategy was adopted for missing data on job category (7% in the mortality study; 5% in the morbidity study) using 20 imputed datasets in both the regression models [25,26] under the reasonable assumption of their missing at random (MAR). A sensitivity analysis made by attributing to different job categories the subjects with missing job information, did not show substantial differences in the main results.

Only causes with at least 10 observed cases were considered for the internal analysis.

The Confidence Intervals (CIs) were estimated by the maximum likelihood method. The 90% level was chosen to limit the use of CIs as significance test thus avoiding the selection of relevant results on the sole basis of “statistical significance” [27].

The statistical analysis was performed using STATA 11.0 software [28].

## Results

Table 1 shows the main descriptive data of the cohort.

**Table 1:** Descriptive data of mortality and morbidity study by job and residence categories

	**Tot.**	**Job category**				**Residence in Gela**	
		***White collars***	***Blue collars***	***Both***^**a**^	**Missing**	***Residents***	***Commuters***
**MORTALITY STUDY**							
N (%)	5,627	1,178 (21)	2,985 (53)	1,072 (19)	392 (7)	2,393 (42.5)	3,234 (57.5)
Person years	178,318	37,092	93,557	35,247	12,422	74,201	104,117
Age at hiring, mean (sd)	26.3 (6)	26.3 (5.6)	26.1 (5.8)	25 (3.9)	31 (9.5)	26.5 (6.3)	26.1 (5.7)
Age at the end of follow-up, median	58.7	58	58.2	60.3	61.7	58.2	59.1
Time since first employment, median	32.5	33	31.7	39.4	34.8	32	33.2
**MORBIDITY STUDY**							
N (%)	5,431	1,141 (21)	3,005 (55.4)	1,005 (18.5)	280 (5.1)	2,419 (44.5)	3,012 (55.5)
Age at the start of follow-up, mean (sd)	55 (10.8)	56 (7.9)	54 (11.7)	56 (10.1)	63 (9.5)	53 (12.1)	57 (9.4)
Time since first employment^b^, median	33.7	34.3	32.6	40.6	37.4	32.6	34.9

^a^ workers who were both blue collar and white collar.

^b^ period from hiring to 01.01.2004.

In the main diseases groups, the SMRs analysis shows that the observed mortality is lower than expected for most of the causes of death (Table 2). A slight increase is observed for primary liver cancer and kidney malignant neoplasms; the increase for asthma is negligible.

**Table 2:** **Standardized Mortality Ratios (SMRs) by selected causes of ***a priori***interest. Reference mortality rates Sicily Region**

**Cause of death**	**ICD - 9**	**Observed**^**a**^	**Expected**	**SMRx100**	**90% CI**
All causes	000-999	563	786.29	72	67-77
Malignant neoplasms	140-208	185.49	246.1	75	66-85
Digestive organs, and peritoneum	150-159	60.78	77.7	78	62-96
Stomach	151	9.43	17.5	54	27-90
Colon, rectum, rectosigmoid junction, and anus	153-154	18.86	19.33	98	60-138
Liver primary	155	12.58	9.31	135	74-209
Respiratory and intrathoracic organs	160-165	66.02	90.33	73	59-90
Trachea bronchus and lung	162	56.59	79	72	56-89
Pleura	163	2.1	2.05	102	17-307
Melanoma of skin	172	1.05	2.49	42	2-191
Other skin	173	1.05	0.84	125	6-565
Genitourinary organs	179-189	18.86	22.73	83	51-117
Bladder	188	6.29	9.8	64	27-121
Kidney	189	6.29	4.65	135	56-255
CNS	191-192; 225	7.34	10.4	71	32-126
Lymphatic and hematopoietic tissue	200-208	15.72	22.67	69	41-102
Hodgkin	200, 202	1.05	2.56	41	2-185
Non-Hodgkin	201	4.19	7.26	58	19-126
Myeloma	203	2.1	2.93	72	12-215
Leukemias	204-208	8.38	9.91	85	40-146
Diseases of the circulatory system	390-459	153	248.4	62	54-70
Diseases of the respiratory system	460-519	29.34	41.01	72	51-96
COPD	490-496	23.05	27.39	84	57-119
Asthma	493	3.14	2.4	131	34-323
Diseases of the digestive system	520-579	36.68	59.7	61	45-80
Diseases of the genitourinary system	580-629	3.14	11.36	28	7-68
Nephritis nephrotic syndrome and nephrosis	580-589	2.1	8.34	25	4-75

^a^See methods for unknown causes of death allocation in “Statistical analysis” paragraph.

Table 3 shows the mortality results by job and residence categories.

**Table 3:** Mortality Rate Ratios (MRRs) by job category (reference *White collars*) and residential classification (reference *Commuters*)

**Cause of death**^**a**^	**ICD - 9**	**Observed**	**MRR**^**b**^**by job category (90% CI) Reference*White collars***		**MRR**^**b**^**by residential classification (90% CI) Reference*Commuters***
			***Both***^**c**^	***Blue collars***	***Residents in Gela***
All causes	000-999	563	0.89 (0.68-1.18)	1.26 (1.01-1.56)	0.89 (0.78-1.03)
Malignant neoplasms	140-208	177	1.49 (0.94-2.36)	1.77 (1.19-2.63)	1.04 (0.81-1.33)
Digestive organs, and peritoneum	150-159	58	1.38 (0.57-3.31)	2.38 (1.15-4.91)	0.72 (0.46-1.14)
Colon, rectum, rectosigmoid junction, and anus	153-154	18	1.66 (0.23-11.8)	3.54 (0.68-18.39)	0.39 (0.15-1.0)
Liver primary	155	12	1.18 (0.32-4.36)	0.50 (0.16-1.64)	1.03 (0.39-2.7)
Respiratory and intrathoracic organs	160-165	63	1.66 (0.77-3.56)	1.71 (0.87-3.35)	1.86 (1.22-2.83)
Trachea bronchus and lung	162	54	1.94 (0.78-4.85)	2.11 (0.96-4.63)	1.71 (1.09-2.69)
Genitourinary organs	179-189	18	1.33 (0.38-4.07)	1.51 (0.51-4.46)	0.16 (0.05-0.56)
Lymphatic and hematopoietic tissue	200-208	15	1.91 (0.45-8.01)	1.45 (0.39-5.37)	1.6 (0.68-3.76)
Diseases of the circulatory system	390-459	146	0.91 (0.53-1.56)	1.25 (0.81-1.93)	0.95 (0.72-1.25)
Diseases of the respiratory system	460-519	28	0.47 (0.72-3.05)	2.45 (0.84-7.15)	0.81 (0.43-1.54)
COPD	490-496	22	0.56 (0.09-3.59)	1.99 (0.59-6.73)	0.58 (0.27-1.25)
Diseases of the digestive system	520-579	35	1.02 (0.4-2.58)	0.9 (0.42-1.9)	0.54 (0.29-1.01)

^a^ Outcomes of a priori interest with at least 10 observed cases.

^b^ MRR estimares are adjusted for age, calendar period, residence category or job category, and time since first employment.

^c^ workers who were both blue collar and white collar in their employment history.

For the majority of the analysed causes, MMRs are higher among *blue collars* than among workers belonged to the *both* job category. Among *blue collars* an increase is detected for all causes, all malignant neoplasms and digestive system malignancies. For colon and lung cancer, disease of respiratory system and chronic obstructive pulmonary diseases (COPD) an increase is suggested. An increased risk with imprecise estimates for the category of all lymphatic and haematopoietic tissue neoplasms is observed in occupational and residential mortality analysis.

MRRs for residents are increased for all malignant neoplasms of the respiratory system and lung cancer, and decreased for genitourinary neoplasms.

Table 4 presents the results for Hospital Discharge Records by job category and residence.

**Table 4:** Hospital Discharge Odds Ratios (HDORs) by job category (reference *White collars*) and residential classification (reference *Commuters*)

**Cause of hospitalization**^**a**^	**ICD - 9 - CM**	**Observed**	**HDOR**^**b**^**by job category ** **(90% CI)** **Reference White collars**		**HDOR**^**b**^**by residential classification** **(90% CI)** **Reference*Commuters***
			***Both***^**c**^	***Blue collars***	***Residents in Gela***
Neoplasms	140-239	420	1.23 (0.91-1.68)	1.33 (1.04-1.72)	1.02 (0.85-1.22)
Malignant neoplasms	140-208	297	1.12 (0.77-1.61)	1.25 (0.93-1.67)	0.95 (0.76-1.18)
Digestive organs, and peritoneum	150-159	69	1.14 (0.56-2.31)	0.99 (0.55-1.78)	1.24 (0.81-1.91)
Colon, rectum, rectosigmoid junction, and anus	153-154	26	1.07 (0.35-3.2)	1.08 (0.44-2.66)	1.39 (0.72-2.67)
Liver primary	155	14	0.69 (0.15-3.05)	0.31 (0.09-1.09)	0.33 (0.09-1.2)
Respiratory and intrathoracic organs	160-165	39	0.78 (0.17-3.46)	2.15 (0.82-5.66)	0.8 (0.42-1.54)
Trachea bronchus and lung	162	31	0.82 (0.18-3.83)	1.76 (0.62-5.01)	0.58 (0.27-1.25)
Other skin	173	26	0.71 (0.22-2.24)	1.11 (0.44-2.81)	0.44 (0.21-0.95)
Genitourinary organs	179-189	110	1.13 (0.65-1.98)	1.23 (0.79-1.92)	0.83 (0.59-1.16)
Bladder	188	33	2.9 (1.1-7.65)	1.25 (0.51-3.06)	0.44 (0.21-0.89)
Kidney	189	12	0.5 (0.07-3.58)	1.13 (0.35-3.67)	2.62 (0.93-7.41)
CNS	191-192; 225	11	0.8 (0.1-6.55)	1.23 (0.31-4.92)	4.95 (1.32-18.47)
Lymphatic and hematopoietic tissue	200-208	23	0.82 (0.22-3.03)	0.85 (0.31-2.35)	0.62 (0.28-1.36)
Non-Hodgkin	200; 202	11	0.96 (0.18-5.27)	0.96 (0.25-3.74)	0.67 (0.23-1.93)
Diseases of the circulatory system	390-459	817	1.25 (0.99-1.57)	1.45 (1.21-1.74)	1.02 (0.89-1.17)
Diseases of the respiratory system	460-519	289	1.08 (0.74-1.57)	1.1 (0.81-1.48)	1.07 (0.87-1.33)
Acute respiratory infections; pneumonia and influenza	460-466; 480-487	97	1.5 (0.83-2.72)	1.26 (0.78-2.03)	1.13 (0.79-1.61)
COPD	490-496	81	1.81 (0.85-3.97)	2.0 (1.0-3.0)	1.4 (0.96-2.06)
Diseases of the digestive system	520-579	636	1.35 (1.06-1.71)	1.17 (0.96-1.43)	1.1 (0.96-1.28)
Diseases of the genitourinary system	580-629	474	1.29 (0.97-1.7)	1.34 (1.06-1.68)	1.23 (1.04-1.45)
Nephritis nephrotic syndrome and nephrosis	580-589	48	2.68 (0.9-7.99)	3.09 (1.15-8.26)	1.31 (0.78-2.18)

^a^ Outcomes of *a priori* interest with at least 10 subjects with an hospital discharge record in the study period.

^b^ HDOR estimates are adjusted for age, residence category or job category, and time since first employment.

^c^ workers who were both blue collar and white collar in their employment history.

An increased risk among *blue collars* for all neoplasms, diseases of circulatory system, COPD, diseases of genitourinary system, nephritis and nephrosis is shown. An increase is suggested for respiratory malignancies.

Among workers in *both* job category an increase in bladder cancer is observed. In the same category an increase for nephritis is suggested.

HDORs among residents are augmented for CNS neoplasms and genitourinary diseases; a decreased risk is shown for skin and bladder cancer. For kidney cancer and COPD an increase is suggested.

## Discussion

### Validity aspects

The procedures adopted for cohort enumeration, follow-up, vital status ascertainment and causes of death coding complied with recommended standards in occupational epidemiology [19]. Based on a previous validity study [29], Sicilian Region’s HDRs were retained of good quality and suitable for this study.

The main limits of the present investigation are the lack of information on individual residential history, occupational and environmental exposures. Sometimes defining occupational exposure in quantitative terms in a retrospective cohort study is not possible [19]. This could be a major problem in analysing the risk for workers in petrochemical complexes, as the one in Gela, where chemical mixtures are common, and their intensity and duration vary over-time [30]. Moreover, when the population is not characterized with respect to specific exposures [31], the risk estimates for some groups could suffer from dilution effect; consequently, identifying risk increases could be more difficult. Finally, in the mortality study of Gela petrochemical workers, the MRR for chronic and long latency diseases might be underestimated because the median age at the end of follow-up is about 59 and 78% of the workers are younger than 65.

The observed deficit in SMR estimates can be mainly attributed to the Healthy Worker Effect (HWE). This effect is common in occupational cohorts and typically characterized by lower mortality from all combined causes of death, cardiovascular, respiratory, and neoplastic diseases [19]. The HWE is a phenomenon detected in different countries and in cohorts experiencing different exposures [32-36]. It has been suggested that the HWE bias can be minimized by the selection of an appropriate comparison such as a working population [32,36] or by analytical methods such as lag time analyses or analyses restricted to newly employed or long term workers [36]. In the present study data limitations did not allow the application of such approaches.

Work and residence categorizations suffer from intrinsic data limitations. As in most occupational cohort studies, data on confounding from lifestyle habits such as smoking, were not available. As for smoking, its prevalence is usually higher in *blue collars* than in *white collars*[37], but this has not been documented in the petrochemical sector where smoking is usually strictly prohibited in several working areas (in Gela plant since 1963). Though, sensitivity analyses have shown that plausible smoking differences will rarely result in a lung cancer “relative risk” greater than 1.5 and will be even lower for diseases that are less strongly associated with smoking [38]. This was also confirmed by several analyses with empirical data in occupational studies [39,40].

For the *both* job category, changes from *blue collar* to *white collar* (95%) or vice versa might not always correspond to a change in the work environment because it could only be a change in the professional level.

The job classification adopted in the present study has consequences for the internal analysis which deserve specific comments. On one hand, an overestimation of MRR in *blue collars* and an underestimation in the *both* job category result from the classification of person-years; on the other hand, an underestimation of risk estimates in *blue collars* cannot be excluded if they had a major risk due to the work environment, because changes from blue collar to white collar might not always correspond to a change in the work environment.

The categorization of residence is intrinsically affected by misclassification, since the likelihood that *commuters* have been resident in Gela is greater than the likelihood that *residents* have been resident elsewhere; this is because the majority of *residents* (70%) were defined only on the basis of place of birth, while all *commuters* were identified using both information on place of birth and applying a mobility model that might include further uncertainties in the classification. As a consequence, if differences in risk are really due to a residential risk in Gela, when comparing *residents* vs. *commuters* underestimation of MRRs and HDORs are expected. For some causes, confounding could be due to residential risky exposures (e.g. urban air pollution) other than to industrial pollution in Gela, but there are no data to infer this point. As far as confounding from lifestyle habits, it is a minor problem in residential analysis because there are no reasons to assume differences between *residents* and *commuters*. Internal residential analyses are adjusted for job categories, thus taking into account, at least in part, possible confounding factors associated with the occupational context. Moreover, confounding for some causes might still occur because of differences in lifestyles between *blue collars* and *white collars*.

Causes selected for analysis in the present study differ in terms of prognosis and duration. Some conditions are highly fatal, others have a more favourable prognosis and may cause repeated hospital admissions. For these reasons, mortality and morbidity data are complementary, and provide a deeper and more realistic description of the population health profile.

The restriction criteria adopted for the morbidity analysis implied the exclusion of subjects deceased before 2003, possibly resulting in a *survival cohort* selected because of good health status. This could lead to an underestimation of the occupational risk, when performing the morbidity analysis, because of major risk observed in the mortality study in the *blue collars* category. The cohort was restricted to workers born in Sicily; the average age was 55 at the start of the morbidity follow-up. This indicated that most of them were likely to be resident active workers in Sicily in the period at study. The number of the subjects possibly lost to the morbidity follow up because of migration outside Sicily, might have been negligible and there are no reasons to believe that the hypothetical losses were differential for the job and residence categories.

The periods of hiring and follow-up considered in the mortality study coincide with the ones of previous analyses [17,18]. Nevertheless, the present investigation has novelties in several elements: restriction only to Sicilian workers, application of a robust mobility model to define residential categories; consideration of all causes of *a priori* interest; computation through a multiple imputation approach of missing data on job categories. Furthermore, in the present study results from the mortality study were considered together with the ones of the morbidity analysis, to disentangle occupational from residential risk. Results of previous mortality studies, whose internal analyses were limited to all causes, all cancer and lung cancer, are consistent with the present ones.

### Study findings

In the light of the observed increased risks, some considerations and comments on specific diseases follow.

The available epidemiological evidence of health effects of residence in the neighbourhood of petrochemical plants is coherently pointing to an increased risk for respiratory diseases, including lung cancer [21]. An augmented risk for lung cancer among petrochemical workers was observed in some occupational cohort studies [21]. The main risk factor for lung cancer is tobacco smoke, but also environmental and occupational factors can contribute to its etiopathogenesis [41]. In the present study, the increase in lung cancer mortality among Gela residents and among blue collars point to a causal link with environmental and occupational exposures respectively. The increased occurrence of lung cancer among *blue collars* is also observed in hospital discharge analysis.

As for lung cancer, the main risk factor for COPD is tobacco smoke, but outdoor pollution and occupational exposure may contribute to the risk of developing the disease [42,43]. COPD mortality is increased in Gela *residents* and in *blue collars*; the latter also show higher HDORs.

Lymphohematopoietic tissue neoplasms showed a 10-15% increased risk for employment in chemical industry [44]. In 1989, the International Agency for Research on Cancer classified occupational exposures in petroleum refining as probably carcinogenic to humans (Group 2A), on the basis of limited evidence for skin cancer and leukaemia [45]. Subsequent petrochemical workers cohort studies confirmed an increased risk for lymphoematopoietic tissue neoplasms [21]. Evidence for residential risk for leukemia is suggested by some studies, while it is scarce and conflicting for other lymphohematopoietic cancers [21]. The present study suggests a moderate increase in mortality, but not in hospital discharges, from lymphohematopoietic neoplasms in the study population.

Because of documented air and water contamination from mercury and other heavy metals in the area of Gela [1], and given the presence of a chlor-alkali unit in the petrochemical plant in the period 1970–1994 [20], renal diseases are also of particular relevance in the present investigation. In fact, heavy metals, namely mercury, cadmium and lead, have an ascertained toxic effect on the kidney [46]. Exposure to mercury might have contributed to the increase in nephritis, nephrotic syndrome and nephrosis among *blue collars*, shown by hospital discharges.

According to Wrensch et al. review of primary brain tumours [47], the question of whether employment in petrochemical, petroleum and oil production industry entails an increased risk remains unresolved. Nevertheless, a relationship between certain parts of the industry and increased risk is documented by the fact that most studies have shown an increased mortality of 20% to 80% [47]. In recent years residential exposure to petrochemicals was investigated in studies with conflicting results [48,49]. The increased risk of CNS cancer in Gela residents shown by hospital discharge analysis might thus be associated with environmental exposures.

For bladder cancer, tobacco smoking is the major established risk factor. Occupational exposure to aromatic amines (for example 2-napthylamine and benzidine) and polycyclic aromatic hydrocarbons are found to increase the risk [50]. Other potential risks are represented by working in dyestuff manufacture, painting, rubber, leather, and aluminium industry. Carcinogens in drinking water (chlorination by-products and arsenic) may increase risk of bladder cancer [51]. It has been estimated that exposure to occupational carcinogens causes up to 25% of all bladder tumours [52]. Some evidence of risk is arising in the petrochemical industry [53]. The increased hospital discharge odds ratio described by the present study among subjects who had been *both* blue and white collars, but not among *blue collars* alone, is not easily interpretable.

## Conclusions

In the light of epidemiological evidence about health risk from petrochemical industries in both occupational and environmental settings, and/or on the basis of information about occupational and residential contamination and health risk in the area of Gela, noteworthy results are shown for lung cancer, respiratory and genitourinary diseases. They support a role of exposures in the occupational and residential settings, the latter due to the local ascertained contamination, in affecting the workers’ health.

These results underline the urgent need of water, soil, air and food-chain monitoring programs, in order to look for active sources of exposure and to define adequate preventive strategies. In particular, as recommended since 1995 [12], an *ad hoc* air pollution program should be implemented.

## Abbreviations

CNS: central nervous system; COPD: chronic obstructive pulmonary disease; HDORs: Hospital Discharge Odds Ratios; HDRs: Hospital Discharge Records; HWE: Healthy Worker Effect; ICD: International Classification of Diseases; IPSs: Italian Polluted Sites; MRRs: Mortality Rate Ratios; SMRs: Standardized Mortality Ratios.

## Competing interests

Roberta Pirastu and Pietro Comba served as technical consultants for the Gela public prosecutor in the first phase of the study.

## Authors’ contributions

RP contributed to all phases of the study: design, data collection, data analysis, manuscript preparation; AZ contributed to design, data collection and management of the morbidity study, and in manuscript preparation; RPirastu contributed to design and data collection of the mortality study, and in manuscript preparation; AC contributed to design and data collection of the morbidity study; GD contributed in discussing study design; SPA contributed in the morbidity data collection and management; SS contributed in discussing study design; PC contributed in study design and manuscript preparation. All authors read and approved the final manuscript.
